# A patient with spontaneous rupture of the esophagus and concomitant gastric cancer whose life was saved: case of report and review of the literature in Japan

**DOI:** 10.1186/1477-7819-9-161

**Published:** 2011-12-06

**Authors:** Nobuhisa Matsuhashi, Narutoshi Nagao, Chihiro Tanaka, Takuo Nishina, Masahiko Kawai, Katsuyuki Kunieda, Hitoshi Iwata

**Affiliations:** 1 Department of Surgery, 4-6-1 Noishiki, Gifu City 500-8717, Japan; 2 Department of Pathology, 4-6-1 Noishiki, Gifu City 500-8717, Japan

**Keywords:** spontaneous rupture of the esophagus, gastric cancer, prediagnoisis

## Abstract

A 71-year-old man suddenly developed abdominal pain and vomiting on drinking soda after a meal, and visited a physician. Cervical subcutaneous and mediastinal emphysemas were observed on CT, and the patient was transferred to the emergency medical center of our hospital on the same day. Esophagography was performed at our department. A ruptured region was identified on the left side of the lower thoracic esophagus, and surgery was emergently performed employing sequential left thoracoabdominal incision. The chest wall was adhered due to inflammation, and large amounts of residual food and sloughing were present in the thoracic cavity and mediastinum. Moreover, necrotic changes were noted in the superior through inferior mediastinum. An about 2-cm rupture site was confirmed on the left side of the lower thoracic esophagus and closed by suture and filling with pediculate omentum. The presence of a tumorous lesion located mainly in the body of the stomach and lymph node enlargement were also diagnosed before surgery, for which gastric and intestinal fistulae were inserted to prepare for the second-stage surgery. The patient was admitted to an ICU after surgery. ARDS and MRSA-induced pneumonia and enteritis concomitantly developed but remitted. Curative surgery for gastric cancer was performed at 40 POD. Spontaneous rupture of the esophagus is relatively rare and that complicated by gastric caner is very rare, with only six cases being reported in Japan. Herein, we report the case.

## Background

Spontaneous rupture of the esophagus is rare, and the life-saving rate is still low. We encountered a very rare case in which rupture occurred due to concomitant gastric cancer. Only 6 cases including this patient have been reported in Japan, and this is the first case report in English. The disease could be preoperatively diagnosed, and the patient could be saved despite the condition being very serious. Herein, we report the case.

## Case presentation

The patient was a 71-year-old male with a chief complaint of abdominal pain. There was no particular past medical history. The patient suddenly developed abdominal pain and vomiting on drinking soda after a meal, and visited a physician. Cervical subcutaneous and mediastinal emphysemas were observed on CT, and the patient was transferred to the emergency medical center of our hospital on the same day. On admission, the height was 168 cm; body weight, 55 kg; BP, 154/76 mmHg; HR, 93/min; respiratory rate, 35/min; and SpO2, 98% (O2 5 L mask). The patient could not hold a supine position due to respiratory distress and pain. On blood testing and chemistry on admission, WBC was 14.5 × 10^3^/ul and CRP was 0.34 mg/dl, showing high levels of inflammatory parameters. BUN was 46 mg/dl and Cr was 4.03 mg/dl, revealing features of prerenal renal failure. PT-INR was 1.35; AT-III, 63%; and FDP, 64 μg/ml. CEA was 11.7 ng/ml, showing a high level.

**Figure 1:**
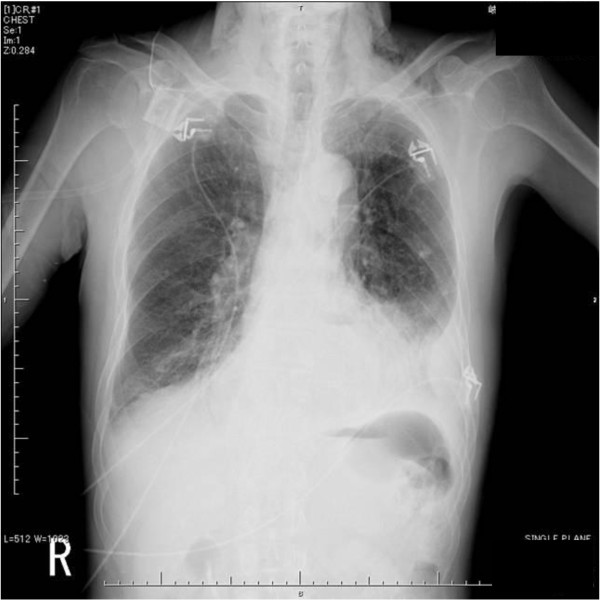
**Plain chest X-ray radiography**. No apparent free air was present, but marked right pleural effusion was observed.

On plain thoracoabdominal X-ray radiography, no apparent free air was present, but marked right pleural effusion was noted (Figure 1).

**Figure 2:**
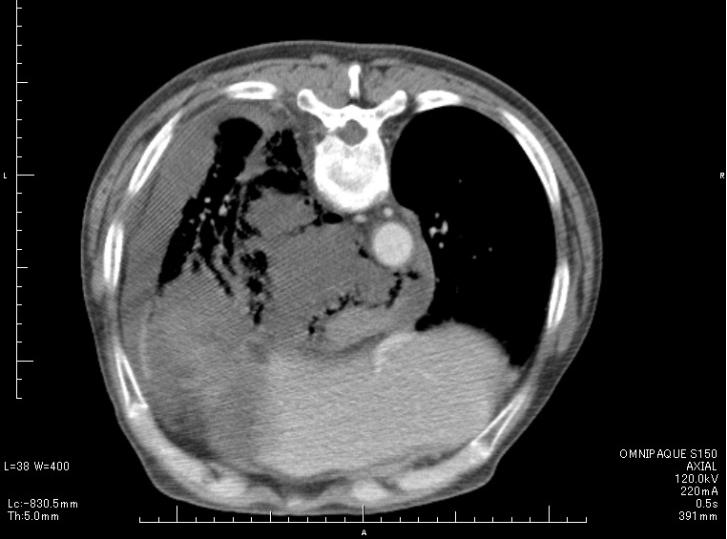
**Chest CT**. Large amounts of residual food and air were observed in the mediastinum, and pleural effusion was also present.

**Figure 3:**
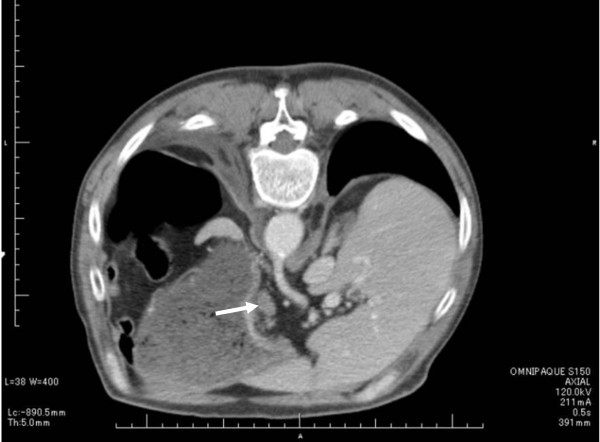
**The stomach was markedly dilated**. The stomach was markedly dilated, and hypertrophy of the antral stomach wall and 15-mm lymph node swelling around it were noted.

On thoracoabdominal CT, marked subcutaneous emphysema was present in the cervical region through the mediastinum, and pleural effusion was also observed (Figures 2). Moreover, the stomach was markedly dilated, and hypertrophy of the antral stomach wall and 15-mm lymph node swelling around it were observed(Figure 3).

The ruptured site was identified on the left side of the lower thoracic esophagus on esophagography and diagnosed as spontaneous rupture of the esophagus. Surgery was emergently performed in consideration of concomitant gastric cancer.

**Figure 4:**
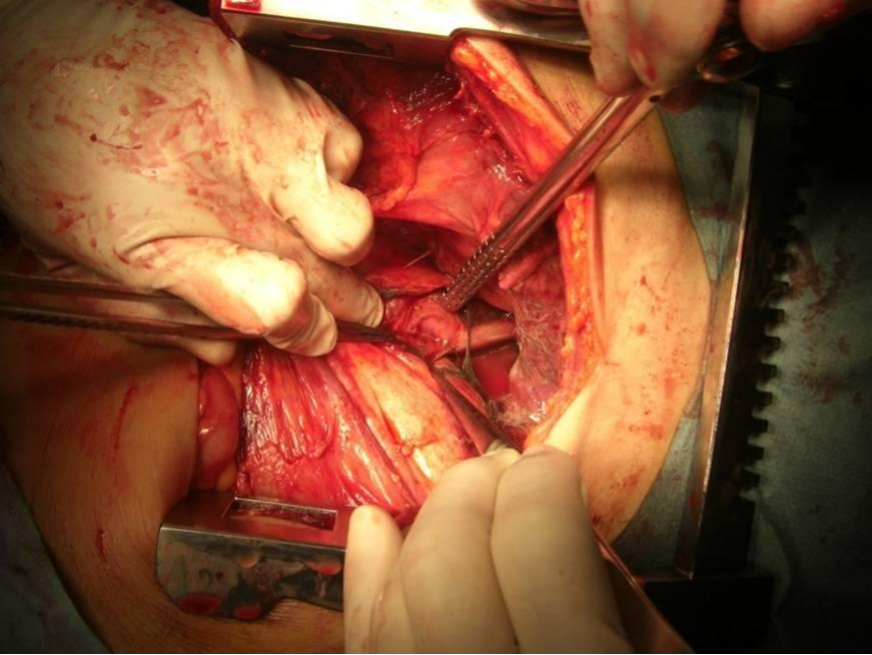
**In surgery employing sequential left thoracoabdominal incision**. In surgery employing sequential left thoracoabdominal incision, the chest wall was adhered due to inflammation, and large amounts of residual food and sloughing were present in the thoracic cavity and mediastinum. Necrotic changes were also noted in the superior through inferior mediastinum.

**Figure 5:**
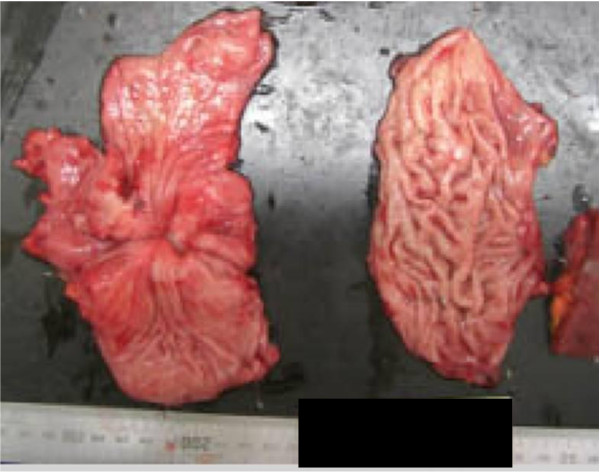
**Total gastrectomy + splenectomy + D2 lymphadenectomy could be performed at 40 POD after the first surgery**. The final pathological diagnosis was pStageIV (TNM classification: T3N1M1) with por 2 and No. 10 lymph node metastasis (Japanese Classification of Gastric Carcinoma).

The first surgery was performed employing sequential left thoracoabdominal incision. The chest wall was adhered due to inflammation, and large amounts of residual food and sloughing were present in the thoracic cavity and mediastinum (Figure 4). Moreover, necrotic changes were noted in the superior through inferior mediastinum. An about 2-cm rupture site was identified on the left side of the lower thoracic esophagus and closed by suture and filling with pediculate omentum. Necrotized substances were removed as much as possible, followed by massive irrigation. The presence of a tumorous lesion mainly in the body of the stomach and lymph node enlargement were also diagnosed before surgery, for which gastric and intestinal fistulae were inserted to prepare for the second-stage surgery (Figure 5).After surgery, the patient was admitted to an ICU, in which concomitant ARDS and sepsis developed. Moreover, MRSA-induced pneumonia and enteritis also developed but remitted. Total gastrectomy + splenectomy + D2 lymphadenectomy could be performed at 40 POD after the first surgery. The final pathological diagnosis was pStageIV (TNM classification: T3N1M1) with por 2 and No. 10 lymph node metastasis (Japanese Classification of Gastric Carcinoma). The postoperative course was favorable, and the patient was alive as of 2 years after surgery.

## Discussion

In spontaneous rupture of the esophagus, a sharp rise in intraesophageal pressure due to vomiting causes full-thickness (mucosa and muscle layer) rupture. Since the disease was initially reported by Boerhaave [1] of Holland in 1724, it is also called Boerhaave syndrome^1)^. In Japan, Yoshida et al. [2]reported it in 1935, and several cases have been reported thereafter. The mortality rate was higher than 40% and gradually decreased, but the outcomes of treatment are still unfavorable compared to other diseases, and the prognosis is particularly poor in patients brought to hospital in a state of shock.

There is a high incidence in men, accounting for 92% of all cases. The onset age peaks in the 40s, and incidences at 30-59 years of age account for 73% of all cases. The rupture site was located in the lower esophagus in 84% and left wall in 67%, and the most frequent inducer was vomiting (64%) followed by other mechanical factors, such as overeating, cough, and trauma, accounting for 19% [3].Regarding the diagnosis, awareness of this disease may lead to early diagnosis. Actually, only about 30% of cases were diagnosed as rupture of the esophagus. When it is suspected, esophagography is performed using an aqueous contrast medium, Gastrografin, and the disease is definitely diagnosed when leakage in the mediastinum or thoracic cavity is observed. As CT has recently spread, noninvasive CT may be useful, especially for patients in a state of shock. MDCT may also be useful for assistive diagnosis of the lesions.

For treatment, surgery and conservative treatment including thoracic cavity drainage are available, but surgical treatment may be the most appropriate [4]. Fukushima et al. reported that the mortality rates after surgical and conservative treatments were 7.7 and 50.0%, respectively, showing unfavorable outcomes in the conservative treatment group [5].

It is still controversial whether laparotomic drainage alone in the mediastinum or thoracotomic drainage should be adopted [6-10].

In our patient, not only rupture of the esophagus but also hypertrophy of the antral stomach wall and lymph node swelling were observed on preoperative MDCT, for which sequential left thoracoabdominal incision (oblique incision) was selected. Gastric (to reduce pressure) and intestinal (for tube nutrition) fistulas were inserted, in addition to drainage, providing useful for postoperative enteric nutrition.

Of cases assumed to be induced by vomiting, duodenal ulcer was the most frequent cause, and gastric ulcer, postgastrectomy disorder, and congenital obstruction are included in the causes. In this patient, in addition to the underlying vulnerability of the esophageal wall and promotion of nausea and vomiting, the pylorus was obstructed by gastric cancer as a local factor of nausea and vomiting. Under these conditions, ingestion of the meal and soda may have rapidly elevated the inner pressure and caused rupture.

This is a valuable case: the 6th case of spontaneous rupture of the esophagus associated with concomitant gastric cancer in Japan [11-14], and the first report in English(Table 1). Since preoperative imaging diagnosis was possible, we report the case including CT and esophagography findings.

**Table 1:** Reported cases of Spontaneous rupture of the esophagus with gastric cancer in Japan

No	Author	Year	Age	Sex	Gastric Cancer	Operative method	Result
1	Yoshila	1981	61	W	L,3type	Distal gastrectomy	better

2	Kishina	1987	53	W	L,3type	Distal gastrectomy	better

3	Itano	1992	65	W	W-L,2type	Distal gastrectomy	better

4	Akioka	1996	62	W	W,O-IIc type	Distal gastrectomy	better

5	Wizutani	1999	47	W	W-L,I+IIa type	Distal gastrectomy	better

6	Our case	2010	71	W	L-W,3type	Total gastrectomy+D2	better

## Conclusion

Spontaneous rupture of the esophagus associated with concomitant gastric cancer are rare.To our knowledge this is the first reported case.

## Consent

Written informed consent was obtained from the patient for publication of this case report and accompanying images.A copy of the written consent is available for review by the Editor-in-Chief of this journal.

## Competing interests

The authors declare that they have no competing interests.

## Authors' contributions

NN and CT took part in the operation. TN,MK and KK performed the literature seach and drafted the manuscript for submission. HI performed histological examination and provided photomicrographs. All authors read and approved the final manuscript.
